# Effect of Ionic Composition on Physicochemical Properties of Mono-Ether Functional Ionic Liquids

**DOI:** 10.3390/molecules24173112

**Published:** 2019-08-27

**Authors:** Hancheng Zhou, Lifei Chen, Zhuo Wei, Yongjuan Lu, Cheng Peng, Bin Zhang, Xiaojuan Zhao, Lan Wu, Yanbin Wang

**Affiliations:** 1 Key Laboratory for Utility of Environment-Friendly Composite Materials and Biomass in Universities of Gansu Province, Department of Chemical Engineering, Northwest Minzu University, Lanzhou 730000, China; 2 College of Pharmacy, Gansu University of Chinese Medicine, Lanzhou 730000, China

**Keywords:** functional ionic liquids, ionic conductivity, heat capacity, phase behavior

## Abstract

Tunable properties prompt the development of different “tailor-made” functional ionic liquids (FILs) for specific tasks. FILs with an ether group are good solvents for many organic compounds and enzymatic reactions. However, ionic composition influences the solubility by affecting the physiochemical properties of these FILs. To address the structure effect, a series of novel FILs with a mono-ether group (ME) based on imidazole were prepared through cationic functionalization and anionic exchange reactions, and characterized by NMR, mass spectroscopy, and Thermogravimetric analysis (TGA). The effect of ionic composition (cationic structure and anions) on density, viscosity, ionic conductivity, electrochemical window, and thermal properties of these ME-FILs were systematically investigated. In general, the viscosity and heat capacity increases with the bigger cationic volume of ME-FILs; in particular, the 2-alkyl substitution of imidazolium enhances the viscosity remarkably, whereas the density and conductivity decrease on the condition of the same [NTf_2_]^−^ anion; For these ME-FILs with the same cations, the density follows the order of [NTf_2_]^−^ > [PF_6_]^−^ > [BF_4_]^−^. The viscosity follows the order of [PF_6_]^−^ > [BF_4_]^−^ > [NTf_2_]^−^. Ion conductivity follows the order of [NTf_2_]^−^ ≈ [BF_4_]^−^ > [PF_6_]^−^. It is noted that the dynamic density has a good linear relationship with the temperature, and the slopes are the same for all ME-FILs. Furthermore, these ME-FILs have broad electrochemical windows and glass transition temperatures in addition to a cold crystallization and a melt temperature for ME-FIL**7**. Therefore, the cationic structure and counter anion affect the physicochemical properties of these ME-FILs together.

## 1. Introduction

During the last 20 years, ionic liquids (ILs), broadly defined as organic salts with a melting point lower than 100 °C, have gained recognition as environmentally benign alternatives to volatile organic solvents and have been applied in catalysis [1,2], separation [3], material synthesis [4,5], and electrochemistry [6,7] because they possess advantageous physicochemical properties including negligible vapour pressure, non-flammability, wide liquid range, and good solvating ability for both organic and inorganic substrates [8,9]. Moreover, the properties of ILs can be modulated by changing the cationic structure or the combination of cation and anion. This tunability has prompted the development of different “tailor-made” ILs for specific tasks, and these functionalized ILs display many often-praised properties [10,11]. For example, Davis et al. first reported a new task-specific IL consisting of an imidazolium cation to which a primary amine moiety is covalently tethered. This novel IL readily and reversibly sequesters CO_2_ at a molar adsorption ratio of 1:2 (mol CO_2_:mol IL) [12,13]. In order to enhance the adsorption ratio, Brennecke et al. introduced a new kind of task-specific ILs, which are entirely comprised of amino acid anions and phosphonium cations, for CO_2_ capture. The adsorption ratio in these cases is nearly equal to 1:1 [14]. Very recently, Dai and Wang synthesized some superbase-derived protic ILs (PILs) and applied these for CO_2_ capture. These anion-functionalized PILs are not only an excellent system for the rapid and reversible capture of CO_2_ with high capacity (more than 1 mol per mol of IL), but they can also act as switchable solvents to simplify the separation processes in organic reactions [15]. Similarly, ILs with chiral groups were utilized as chiral organocatalysts and chiral ligands in asymmetric synthesis [16,17]. ILs with terminal thiol groups were used to stabilize nano-Au in the process of particle fabrication [18,19]. The employment of the ILs with metal ion-ligating groups in the extraction of metal ions from wastewater has also been extensively explored [20]. Indeed, ILs with different functional groups can meet specific requirements for many potential applications [21,22].

Ether-functionalized ILs, having covalently tethered ether functional groups on the cation, were synthesized firstly by Kimizuka for dissolving carbohydrates such as β-d-glucose, α-cyclodextrin, glucose oxidase, and a glycosylated protein [23,24]. Later, Salunkhe extended the application of 1-methoxyethyl-3-methyl imidazolium methanesulfonate IL ([MOEMIm][OMs]) to nucleoside chemistry, and the good solubility of nucleosides in the IL facilitated the synthesis reaction [25]. Almost at the same time, Itoh and Zhao reported that the poly(oxyethylene) alkyl-functionalized ILs are good additives, as well as solvents for enzymatic reactions due to their good biocompatibility [26,27,28]. Recently, we reported that mono-ether functionalized ILs (ME-FILs) have good biocompatibility for lipase in catalyzing the kinetic resolution of secondary alcohols [29]. However, to the best of our knowledge, there is no systematic investigation of the physicochemical properties of ether-functionalized ILs as a function of anion or ether group position.

**Scheme 1:**
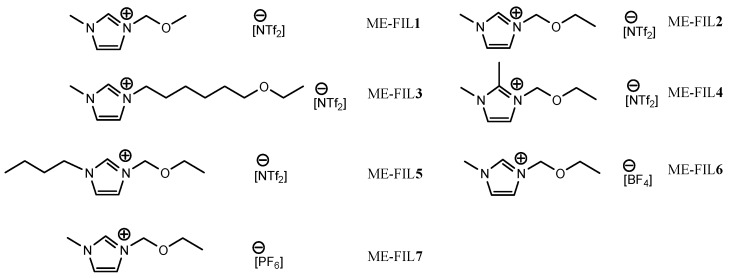
Structures of synthesized mono-ether functional ionic liquids (ME-FILs).

Herein, we report our recently developed series of imidazolium-based ME-FILs containing bis(trifluoromethyl-sulfonyl)imide ([NTf_2_]^−^), ([BF_4_]^−^) and ([PF_6_]^−^) anions as summarized in Scheme 1, and the effect of cationic structure and counter anions on physicochemical properties such as density, viscosity, electrical window, ionic conductivity, thermal stability and phase behavior of these ME-FILs were investigated and discussed in detail.

## 2. Results and Discussion

ME-FILs were synthesized through mono-ether functionalized cation formation and anion exchange and purified as reported previously [30,31,32]. The structures of ME-FILs were characterized by ^1^H, ^19^F, and ^13^C-NMR and mass spectroscopy. The single decomposition peak in the TGA profiles implies that the synthesized ME-FILs were not mixed compounds, which coincides with NMR data and mass spectra (see Supplementary Figures S2, S3 and S4). Yields of 84−97% were obtained for all ME-FILs, and the water concentration were lower than 20 ppm for most of them except ME-FIL**1** (32 ppm) and ME-FIL7(<100 ppm), and ME-FIL**6** is moisture unstable. 

### 2.1. Density and Viscosity

**Figure 1:**
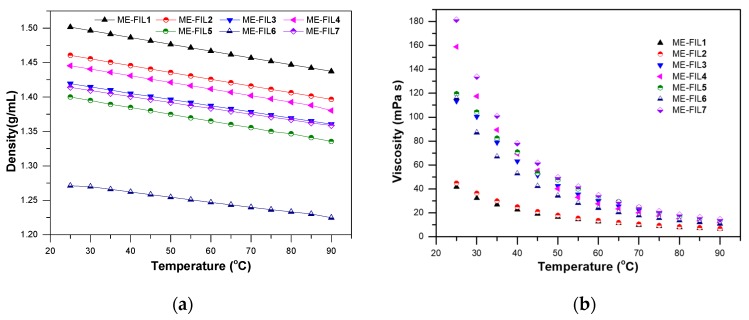
Temperature dependence of the physicochemical property of ME-FILs. (**a**) Dynamic density; (**b**) dynamic viscosity.

The density and viscosity of these ME-FILs at 25 °C are listed in Table 1. It can be seen that both cations and anions have a significant effect on the physicochemical properties. For example, ME-FILs containing [NTf_2_]^−^ possess the highest density compared to [BF_4_]^−^ and [PF_6_]^−^ salts provided that the cationic structures are the same, and the lowest viscosity among ME-FIL**2**, ME-FIL**6**, and ME-FIL**7**. Bearing the same anion ([NTf_2_]^−^), their densities follow the sequence (at 25 °C) of **1** > **2** > **4** > **3** > **5**, whereas the viscosity is nearly in the reverse order of **4** > **3** > **5** > **2** > **1**. The abnormal higher viscosity of ME-FIL**4** compared to ME-FIL**3** and ME-FIL**5** suggests that the inhibition towards the rotational freedom of the main chain is caused by a methyl substituent at the 2-position of imidazolium [33,34,35]. A similar trend was also observed for ME-FIL**2** and ME-FIL**5**; i.e. that a longer alkyl chain at the 3-position of imidazolium results in lower density and higher viscosity. Similarly, a longer ether chain at the 1-position of imidazolium of ME-FIL**3** leads to a higher viscosity (113 cP) than ME-FIL**2** (44.9 cP). In comparison with non-ether-functionalized ILs with the same structure, it is noteworthy that the introduction of a mono-ether group leads to a definite reduction in viscosity (µ(ME-FIL**2**) = 44.9 cP vs. µ([BMIm][NTf_2_]) = 59.82 cP) [36], which is encouraging for a variety of applications. Furthermore, Figure 1a shows the dynamic density of ME-FILs with typical structures. With the increase of temperature, an almost linear decrease of density was observed for all studied ME-FILs. In addition, despite structural differences, they have nearly the same slope, indicating an approximate rate of decline of density for ME-FILs in the range of tested temperature, and these linear lines fit to the following equation:

*d*_T_ = −1.007 × 10^−3^T/K + C (1)

where *d*_T_ is the dynamic density (g/mL), T (K) is the temperature, and C is a constant (in which C is the difference for different ME-FILs).

The dynamic viscosity of ME-FILs is depicted in Figure 1b. It is obvious that the viscosities of ME-FIL**1** and ME-FIL**2** are far lower than others at 25 °C. However, the wide gaps gradually diminished after the temperature was elevated in the following processes. Finally, the viscosity differences become much less significant at 90 °C, suggesting similar intra- or inter- molecular factors control viscosity in the ME-FILs at higher temperatures. ME-FIL**1** and ME-FIL**2** show markedly less temperature dependence than the other ME-FILs, indicating good temperature stability.

### 2.2. Electrochemical Window

**Figure 2:**
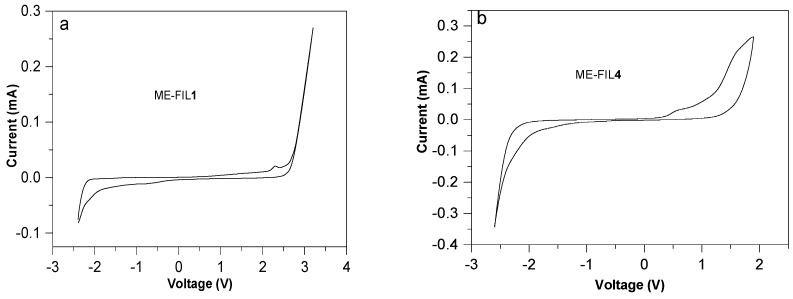
Typical cycle voltammograms of ME-FILs. (**a**) ME-FIL**1**; (**b**) ME-FIL**4**.

The electrochemical stability of all of these ME-FILs was analyzed with cyclic voltammetry at 25 °C (Table 1). They show wide electrochemical windows, comparable to that of 1-alkyl-2-methyl pyrrolinium bis(trifluoromethanesulfonyl)amide (5.0 V) [37]. It is noteworthy that ME-FIL**1** and ME-FIL**2** are electrochemically stable in the potential range from −2.3 V to 2.4 V and −2.5 V to 2.5 V versus the Ag/AgCl electrode, respectively, indicating potential electrolyte applications comparable to non-ether FIL such as [BMIm][NTf_2_] (4.2 V) [38]. In comparison with ME-FIL**1** and ME-FIL**2**, the narrower electrochemical stability window of ME-FIL**3** may result from the unremoved low content of Cl^−^ and Na^+^. Figure 2b depicts the electrochemical behavior of ME-FIL**4**. It is obvious that an irreversible oxidation can be observed at 1.5 V, and a reduction peak can be seen at −2.12 V. Therefore, ME-FIL**4** is stable in the potential range from −2.10 V to 1.25 V versus the Ag/AgCl electrode. Figure 2a is a typical cyclic voltammogram of ME-FIL**1**, and the electrochemical window is about 5.0 V.

### 2.3. Ionic Conductivity

**Figure 3:**
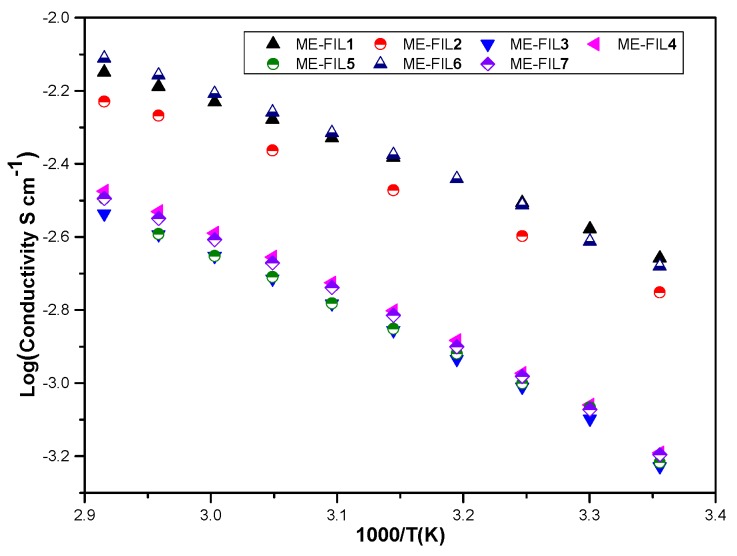
Arrhenius plots of ion conductivity for typical ME-FILs.

The dynamic conductivities have been measured in the temperature range from 25 °C to 70 °C for ME-FIL**1**–**5**, which have a typical group in the 1-, 2-, and 3-position of imidazolium cation, and ME-FIL**6**–**7** with different anions (Figure 3). It can be seen that these plots show typical Arrhenius behaviors, suggesting an ionic association within the ME-FILs. Table 1 indicates that ME-FIL**1** possesses the highest ionic conductivity (0.26 S/m) at 25 °C among all the examined ME-FILs. This might be attributed to the smaller size of the cation and lower viscosity, resulting in the higher rate of ionic mobility. In comparison with ME-FIL**3** (0.073 S/m), the slower mobility of the bigger cation may result in lower conductivity (0.064 S/m) of ME-FIL**5**, although with lower viscosity. However, high space resistance enhances the viscosity dramatically, resulting in the lowest conductivity of ME-FIL**4** (0.059 S/m) among the ME-FILs with an [NTf_2_]^−^ anion, although the cationic size is not the largest. The effect of the anion on ionic conductivity was compared among ME-FIL**2**, ME-FIL**6**, and ME-FIL**7**. ME-FIL**2** combining the [NTf_2_]^−^ anion has a higher ionic conductivity (0.22 S/m), which is close to the value of ME-FIL**6** with a [BF_4_]^−^ anion (0.21 S/m), than ME-IL**7** with a [PF_6_]^−^ anion (0.064 S/m), and lower than the value (0.406 S/m) of non-ether-functionalized ILs [BMIm][NTf_2_] [39]. This significant difference in ionic conductivity caused by anions may be related to the different hydrogen bond interactions between the anion and cation of ME-FILs, and the stronger interaction limit, the freeer the mobility of both the cation and anion [40]. Therefore, both cations and anions play important roles in determining the ionic conductivity of ME-FILs.

### 2.4. Thermal Stability

**Figure 4:**
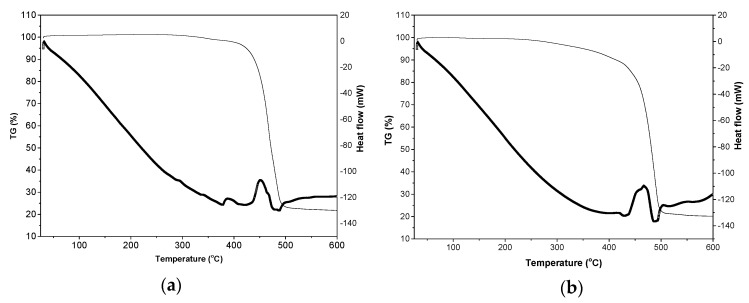
Typical TGA curves of ME-FILs. (**a**) ME-FIL**1**; (**b**) ME-FIL**4**.

Thermogravimetric analysis was carried out to analyze the thermal stability of ME-FIL**1**–**7**, and the decomposition temperatures are listed in Table 2. The results show that these ME-FILs are all stable to at least 250 °C, while ME-FIL**1** only shows the onset of decomposition at 441 °C. ILs with the same anion ([NTf_2_]^−^) showed a general trend that a longer alkyl chain substitution (at the 1 or 3 positions) on the imidazolium ring tend to have lower thermal stability than shorter alkyl chain substituted analogues. Moreover, anions also play a significant role in affecting the thermal stability of ME-FILs in the order of decomposition temperatures: [NTf_2_]^−^ > [PF_6_]^−^ > [BF_4_]^−^, which is consistent with the density trend (ME-FIL**2** > ME-FIL**7** > ME-FIL**6**), and the thermal decomposition temperature *T*_d_ = 430 °C of ME-FIL**2** is near to [BMIm][NTf_2_] (421 °C) [41,42], which has a similar structure to ME-FIL**2** but without ether functionalization. The higher thermal stability of ME-FILs with [NTf_2_]^−^ may be attributed to stronger Van Der Waals forces between the cation and anion [43]. Figure 4 showed the typical TGA curves of ME-FILs.

### 2.5. Heat Capacity and Heat Storage Density

Heat capacities (*C*_p_) are vital for the design of physicochemical processing and reaction units, and the application of ILs as thermal fluids. In order to determine the *C*_p_ of a material, a three-step method is necessary [44]. Therefore, the heat capacity scans of these ME-FILs were completed from −40 °C to 40 °C (see Supplementary Figure S6), a region in which there is no obvious phase change. The thermal analysis data are presented in Table 3.

As can be seen from Table 3, the heat capacity values of ME-FILs with the [NTf_2_]^−^ anion increase as the ether chain length of the cation increases. For example, the heat capacity of ME-FIL**1**, which has the shortest ether chain on the cation among all tested ME-FILs, is much lower than that of ME-FIL**3** (488.4 J·K^−1^mol^−1^ < 668.6 J·K^−1^mol^−1^) at 25 °C (see Supplementary Figure S6). Similarly, the anions affect the *C*_p_ of ME-FILs by the same order as other ILs previously reported in the literature [45,46,47]. For example, the *C*_p_ values of ME-FIL**2**, ME-FIL**6**, and ME-FIL**7** (which have the same cation) decrease in the order of [NTf_2_]^−^ > [PF_6_]^−^ > [BF_4_]^−^ at 25 °C. The high sensible heat storage densities indicate that these ME-FILs could potentially be used as excellent heat transfer fluids.

### 2.6. Phase Behavior

**Figure 5:**
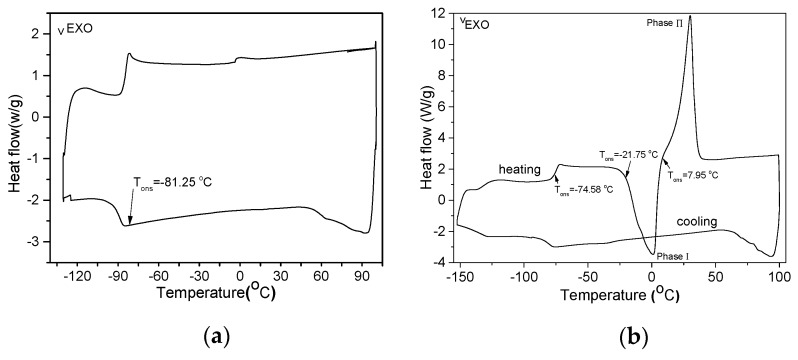
Typical DSC curves of ME-FILs. (**a**) ME-FIL**2**; (**b**) ME-FIL**7**. ^v^EXO represents the exothermic direction; T_ons_ represents the onset of heat capacity change for *T*_g_, or the onset of an endothermic change for *T*_m,_ or the onset of an exothermic change for *T*_cc_.

The phase behavior of ME-FILs was further investigated by differential scanning calorimetry (DSC). From the DSC traces (Figure 5 and Supplementary Figure S22), we can see that the ME-FILs have similar glass transition temperatures (*T*_g_) between −68 °C and −81 °C, which may be related to their similar intra- or inter- molecular interactions. It is obvious that the smaller the size of the cation, the lower the *T*_g_ for the ME-FILs containing a [NTf_2_]^−^ anion. This tendency is the same for viscosity. A heat capacity change corresponding with the glass transition for all of ME-FILs with a [NTf_2_]^−^ anion can be seen during either cooling from 100 °C to −130 °C or heating from −130 °C to 100 °C. ME-FIL**6** displays a heat capacity change at −76.71 °C on heating, and the glass transition temperature is −75.46 °C (Table 2). ME-FIL**7**, however, shows a type of behavior that differs from the above, and its thermal scan is presented in Figure 5b. It can be seen that this IL does not show a tendency to crystallize on cooling; however, the glass transition, cold crystallization, and melting temperatures can be inferred on heating. As expected, the glass transition temperature of ME-FIL**7** (−74.58 °C) is near to that of ME-FIL**2** (−81.25 °C) (Table 2), which has the same cation as ME-FIL**6** and ME-FIL**7**. Therefore, both cations and anions affect the phase behaviors of ME-FILs.

## 3. Materials and Methods

### 3.1. Materials

The compounds 2-bromoethyl methyl ether, 2-bromoethyl ethyl ether, 1-bromo-6-chlorohexane (99%), lithium bis(trifluoromethylsulfonyl)imide (98+%), and silver bis(trifluoromethylsulfonyl)imide (97+%) were purchased from Sigma Aldrich, China. Ethanol (AR), methylimidazole (AR), 1,2-dimethylimidazole (AR), butylimidazole (AR), sodium tetrafluoroborate (AR), ammonium hexafluorophosphate (AR) and other chemicals (AR) were purchased from Fluka. 

### 3.2. Methods

NMR measurements were recorded on a Brüker AV-400 Fourier transform NMR spectrometer using an inner capillary filled with CD3OD for 1H, 13C, and 19F-NMR. Chemical shifts were reported in parts per million (ppm, δ). High resolution mass spectra were recorded on a GC model 6890 N (Agilent Technologies, Waldbrom, Germay) fitted with a split/splitless injector and equipped with a MSD model 5975B (Agilent Technilogies, Tokyo, Japan). IL/methanol solutions (1 µL) were injected in each case automatically by an Autosampler model 7683 (Agilent).

Density measurements were performed using an Anton Paar DMA 5000 density meter with an uncertainty of ±0.00005 g·cm^−3^ for all ME-FILs from room temperature (25 °C) to 70 °C. The viscosity was measured using an Anton PaarAMVn viscometer (Austria) for all ME-FILs from 25 °C to 70 °C.

The ionic conductivity was evaluated using alternating current (AC) impedance spectroscopy in the frequency range of 0.1 Hz to 10 MHz using a dip cell. The measurements were performed with a frequency response analyzer (Solartron1296, Britain) driven by Solectron impedance measurement software version 3.2.0. For all ME-FILs, the temperature range was 25 °C to 70 °C. 

The cyclic voltammetry curve was detected by using a CHI 660A Electrochemical Work Station at 25 °C in the glove box. A 3 mm diameter platinum working electrode, a platinum wire counter electrode, and an Ag/AgCl reference electrode were used in detection, in which the reference electrode consisted of a silver electrode immersed in [BMIm][NTf_2_] saturated with silver bis(trifluoromethylsulfonyl)imide, and was separated from the tested ME-FILs by ceramic chips [48].

Thermogravimetric analysis (TGA) was performed with a Simultaneous Thermal Analysis (STA) 409EP. The samples for TGA were placed in an aluminum crucible. Thermal analysis and temperature-dependent mass changes were examined in the range of 30 °C to 600 °C. The thermal decomposition temperature (*T*_d_) was recorded with 10% mass loss of ME-FILs with a scan rate of 10 °C/min under N_2_ atmosphere.

The phase transitions of all ME-FILs were performed on a thermal analysis (TA) Instruments DSC2010 differential scanning calorimeter in the temperature range of −130 °C to 100 °C at a scan rate of 10 °C/min. In each performance, about 5 mg ME-FILs was sealed in an aluminum pan to test. The melting temperature (*T*_m_) was taken as the onset of an endothermic peak on heating. The glass temperature (*T*_g_) was taken as the onset of heat capacity change; the cold crystallization temperature (*T*_cc_) was taken as the onset of an exothermic peak on heating from a subcooled liquid state to a crystalline solid state.

## 4. Materials and Methods

### 4.1. General Procedures for the Preparation of ME-FILs

The imidazolium-based ME-FILs were prepared according to the previously reported procedure [30,31] with little modification and their abbreviations are summarized in Scheme 1. Except for ME-FIL**3**, all others were prepared by consecutive neutralization and anion exchange reactions (see Supplementary Figure S1). Taking ME-FIL**3** as an example, the detailed synthesis process was as follows: under vigorous stirring, 0.50 g sodium metal was consecutively added into a 50 mL flask containing 20 mL anhydrous ethanol. The mixture was stirred at room temperature until no further hydrogen was released. The excess ethanol was removed by evaporation. Then, 3.97 g (0.02 mol) 1-bromo-6-chlorohexane was added into the above sodium ethoxide under vigorous stirring and refluxing conditions. After the reaction was completed, the solid by-product, sodium bromide, was removed by filtration. The filtrate was collected and transferred into a 100 mL flask. Afterward, 1.64 g 1-methylimidazole (0.02 mol) was added into the filtrate. The mixture was stirred at 70 °C for 8 h, and the bottom product was separated from the upper residue. After separation, 100 mL of an aqueous solution containing 5.74 g (0.02 mol) lithium bis(trifluoromethylsulfonyl)imide was added to the product, followed by agitation for 4 h at room temperature. The mixture was then kept at room temperature until the interface between the IL and the water phase was observed. The IL phase was collected and washed several times with distilled water, followed by removal of water in vacuum at 120 °C for at least 24 h, then treated with anhydrous CaCl_2_ pellets, which produced 9.34 g ME-FIL3 (94.0 % yield). 

### 4.2. Water Content Quantification

The water content in all ME-FILs was quantified before each experiment by using Karl–Fischer coulometric titration (C10SX from Mettler-Toledo, Switzerland). Before the titration, each ME-FIL was rotary evaporated in a vacuum at 120 °C for at least 24 h and treated with anhydrous CaCl_2_ pellets. Then, 3 g ILs were used as titration samples (H_2_O concentration detection limit = 4 ppm/0.3 mM).

## 5. Conclusions

Mono-ether functionalized ionic liquids with different structures have been successfully prepared through consecutive reactions of cationic functionalization and anionic exchange. These ME-FILs possess low viscosity, broad electrochemical windows, good ionic conductivity, and high thermal stability. Cationic structure and counter anions affect the physicochemical properties of these ME-FILs, and the good biocompatibility highlights their use as a potential medium or additives for biochemical reactions with a wide range of applications [26,27,28,29,30,31].

## Figures and Tables

**Table 1:** Density, viscosity, electrochemical window, and ionic conductivity of ME-FILs at 25 °C.

ME-FIL	Density (g/mL)	Viscosity (cP)	Electrochemical Window (V)	Ionic Conductivity (S/m)
1	1.501	41.8	4.7	2.6 × 10^−1^
2	1.459	44.9	5.0	2.2 × 10^−1^
3	1.420	113	3.9	7.3 × 10^−2^
4	1.445	158	4.8	5.9 × 10^−2^
5	1.400	109	4.8	6.4 × 10^−2^
6	1.270	116	4.5	2.1 × 10^−1^
7	1.413	181	4.6	6.4 × 10^−2^

**Table 2:** Thermal stability and phase behavior of ME-FILs.

ME-FIL	*T*_d_^a^(°C)	*T*_cc_^b^(°C)	*T_g_*^c^(°C)/Δ*C*_p_^d^ (J/g/°C)	*T*_m_^e^ (°C)
**1**	441		−81.53/0.15	
**2**	430		−81.25/0.24	
**3**	307		−78.64/0.77	
**4**	410		−68.73/1.0	
**5**	298		−76.18/0.89	
**6**	269		−75.61/0.18	
**7**	330	−21.75	−74.58/0.16	7.95

^a^ decomposition temperature; ^b^ cold crystallization temperature; ^c^ glass transition temperature; ^d^ Δ*C*_p_ is the heat capacity change during the glass transition; ^e^ melting temperature.

**Table 3:** Heat capacity (*C*_p_) and heat storage density (*D*_h_) for some ILs.

ILs	ME-FIL1	ME-FIL2	ME-FIL3	ME-FIL4	ME-FIL5	[BMIm][BF_4_] ^b^
*C*_p_ (J/g/°C)	1.2	1.24	1.37	1.25	1.39	1.60
*D*_h_^a^	180.0	181.4	194.5	178.8	194.6	192.0

^a^*D*_h_ = ρ·*C*_p_·ΔT, ΔT = 100; ^b^ Ref [46].

## Supplementary Materials

The following are available online at https://www.mdpi.com/1420-3049/24/17/3112/s1, Figures S1–S17: ^1^H, ^13^C, and ^19^F spectra of ME-FIL**1, 2,3,4,6,** and **7,** respectively; Figures S18–S21: Cationic and anionic Mass spectra of typical ME-FIL**1** and **3**, respectively; Figure S22: DSC curves of MEF-IL**1**, **3**, and **4**; Figure S23: CV curves of MEF-IL**2** and **3**; Figure S24: “Three-step” method for the determination of heat capacities. Table S1: Water content of different ME-FILs.
