# Supplementary material for: Effect of Ionic Composition on Physicochemical Properties of Mono-Ether Functional Ionic Liquids
Source: Molecules. 2019 Aug 27;24(17):3112. doi: 10.3390/molecules24173112 (PMC6749295; doi:10.3390/molecules24173112)
Supplement: Supplementary file 1 [file molecules-24-03112-s001.pdf]

Electronic supplementary material for

Effect of Ionic Composition on Physicochemical Properties of Mono-Ether Functional Ionic Liquids

Hancheng Zhou[1,*], Lifei Chen[1], Zhuo Wei[1], Yongjuan Lu[1,*], Cheng Peng, Bin Zhang, Xiaojuan Zhao[2], Lan Wu[1,*] Yanbin Wang[1]

Contents

S1. Synthesis of MEF-ILs…………………………………………………………………………3

S2. NMR spectral data of some representative ME-FILs……………………………………………4

S3. NMR spectra of some representative ME-FILs…………………………………………………6

S4. Mass Spectra of the typical ME-FILs....................…………………………………….....................15

S5. Water content of the ME-FILs...................………………………………………..…………… 17

S6. DSC curves of the representative ME-FILs…………………………………...…………… 18

S7. Cyclic voltammogram of ME-FILs………………………...19

S8. Calculation for heat capacity of ME-FILs………………………………………………………. 20

S1. Synthesis of ME-FILs

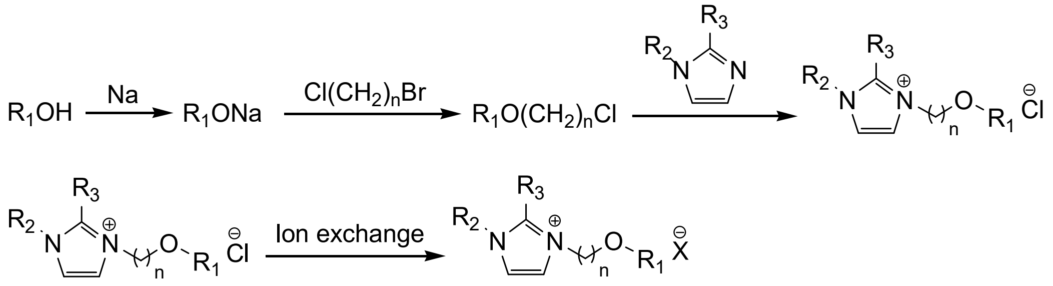

Scheme 1. Synthesis of MEF-ILs.

X= NTf_2 [bis(trifluoromethylsulfonyl)amine], (1) n=1, R_1=CH_3, R_2=CH_3, R_3=H: ME-FIL**1**, [$MImCH_2OCH_3$][NTf_2]; R_1=C_2H_5, MEF-IL**2**, [$MImCH_2OC_2H_5$][NTf_2]; R_1=C_2H_5, R_3=CH_3, MEF-IL**4**, [$MMImCH_2OC_2H_5$][NTf_2]; R_1=C_2H_5, R_2=C_4H_9, MEF-IL**5**, [$BMImCH_2OC_2H_5$][NTf_2];. (2) n=6, R_1=C_2H_5, R_2=CH_3, R_3=H, MEF-IL**3**, [$MIm(CH_2)_6OC_2H_5$][NTf_2].

X=BF_4, n=1, R_1=C_2H_5, R_2=CH_3, R_3=H, MEF-IL**6**, [$MIm(CH_2)_2OC_2H_5$][BF_4].

X= PF_6, n=1, R_1=C_2H_5, R_2=CH_3, R_3=H, MEF-IL**7**, [$MIm(CH_2)_2OC_2H_5$][PF_6].

Example for synthesis of ME-FIL1

Under reflux condition, 2.10 g 1-methylimidazole (0.025 mol) was dissolved in 3.60 g 2-bromoethyl methyl ether (0.026 mol) and stirred for 3 h at 70 ºC. A viscous liquid was obtained after evaporated the resultant mixture at 80 ºC under vacuum and transferred into an aqueous solution of lithium bis(trifluoromethanesulfonyl)imide (0.025 mol). After agitation for 2 h at room temperature, the mixture separated automatically into two layers. The bottom product layer was separated and washed with distilled water (20 mL×4), then rotary evaporated in vacuum, and 9.66 g ME-FIL**1** was obtained, the yield attained to 92%.

ME-FIL**2**, **4**, and **5** were prepared as the aforementioned method except that 2-bromoethyl ethyl ether was used for synthesis of ME-FIL**2**, 1,2-dimethylimidazole, and butylimidazole were used, respectively, for synthesis of **4** and **5**. The prepared procedures for ME-FIL**6** and **7** were the same as **2** except that the anion exchange in the second step.

S2. NMR spectral data of some representative ME-FILs

ME-FIL**1**: *1-methoxymethylene-3-methylimidazolium bis(trifluoromethylsulfonyl)imide,* ([$MImCH_2OCH_3$][NTf_2]): ^{1}H NMR (CD_3OD): 2.08 (s, 1H), 3.28 (s, 3H), 3.63 (s, 3H), 3.85 (t, 2H), 4.25 (t, 2H), 7.27 (d, 1H), 7.34 (d, 1H), 8.51 (s, 1H). ^{13}C NMR (CD_3OD): 36.1, 49.7, 58.6, 76.6, 121.3 (q, CF_3, J), 123.3, 136.1. ^{19}F NMR (CD_3OD):-79.3(C-F).

ME-FIL**2**: *1-ethoxymethylene-3-methylimidazolium bis(trifluoromethylsulfonyl)imide,* ([$MImCH_2OC_2H_5$][NTf_2]): ([$MImCH_2OC_2H_5$][NTf_2]): ^{1}H NMR (CD_3OD): 1.16(t, 3H), 1.92 (s, 1H), 3.49 (t, 2H), 3.73 (s, 3H), 3.92 (t, 2H),4.32(t,2H), 7.31 (d, 1H), 7.41 (d, 1H), 8.62(d, 1H). ^{13}C NMR (CD_3OD): 14.7, 36.2, 50.0, 67.7, 76.7, 121.4 (q, CF_3, J), 123.2, 136.1. ^{19}F NMR (CD_3OD):-79.2(C-F).

ME-FIL**3**: *1-ethoxyhexamethylene-3-methylimidazolium bis(trifluoromethylsulfonyl)imide,* ([$MIm(CH_2)_6OC_2H_5$][NTf_2]): ^{1}H NMR (CD_3OD): 1.13 (t, 3H), 1.29 (quint, 4H), 1.50 (quint, 2H), 1.80 (quint, 2H), 3.32 (t, 2H), 3.39 (q, 2H), 3.88 (s, 3H), 4.11 (t, 2H), 7.19 (d, 1H), 7.32(d, 1H), 8.76(s, 1H). ^{13}C NMR (CD_3OD): 15.2, 25.4, 26.1, 28.0, 29.8, 33.9, 50.2, 66.0, 70.7, 121.4 (q, CF_3, J), 123.6, 136.3. ^{19}F NMR (CD_3OD):-79.0(C-F).

ME-FIL**4**: *1-ethoxymethylene-2,3-dimethylimidazolium bis(trifluoromethylsulfonyl) imide,* ([$MMImCH_2OC_2H_5$][NTf_2]): ^{1}H NMR (CD_3OD): 1.04 (t, 3H), 2.52 (s, 3H), 3.36 (q, 2H), 3.50 (s, 3H), 3.70 (t, 2H), 4.15 (t, 2H), 6.75 (d, 1H), 7.15 (d, 1H). ^{13}C NMR (CD_3OD): 5.6, 14.7, 32.9, 48.9, 66.7, 77.1, 114.8, 121.4, 125.2 (q, CF_3, J), 144.7. ^{19}F NMR (CD_3OD):-79.2(C-F).

ME-FIL**5**: *1-ethoxymethylene-3-butylimidazolium bis(trifluoromethylsulfonyl) imide,* ([$BMImCH_2OC_2H_5$][NTf_2]): ^{1}H NMR (CD_3OD): 0.99 (t, 3H), 1.14 (t, 3H), 1.31 (m, 2H), 1.80 (quint, 2H), 3.38 (q, 2H), 3.72 (t, 2H), 3.87(t,2H), 3.92(t,2H), 7.01 (d, 2H), 7.28 (s, 1H). ^{13}C NMR (CD_3OD): 13.9, 15.2, 20.0, 34.8, 49.7, 53.8, 65.7, 72.8, 117.4 (q, CF_3, J),141.3, 144.6. ^{19}F NMR (CD_3OD): -79.6(C-F).

ME-FIL**6**: *1-ethoxymethylene-3-methylimidazolium tetrafluoroborate,* ([$MImCH_2OC_2H_5$][BF_4]):

([$MImCH_2OC_2H_5$][BF_4]): ^{1}H NMR (CD_3OD): 1.16(t, 3H), 1.92 (s, 1H), 3.49 (t, 2H), 3.73 (s, 3H), 3.92 (t, 2H),4.32(t,2H), 7.31 (d, 1H), 7.41 (d, 1H), 8.62(d, 1H). ^{13}C NMR (CD_3OD): 14.7, 36.2, 50.0, 67.7, 76.7, 123.2, 136.1. ^{19}F NMR (CD_3OD):-151.1(B-F).

MEF-IL**7**: *1-ethoxymethylene-3-methylimidazolium hexafluorophosphate,* ([$MImCH_2OC_2H_5$][PF_6]):

([$MImCH_2OC_2H_5$][PF_6]): 1.16(t, 3H), 1.92 (s, 1H), 3.49 (t, 2H), 3.73 (s, 3H), 3.92 (t, 2H),4.32(t,2H), 7.31 (d, 1H), 7.41 (d, 1H), 8.62(d, 1H). ^{13}C NMR (CD_3OD): 14.7, 36.2, 50.0, 67.7, 76.7, 123.2, 136.1. ^{19}F NMR (CD_3OD):-73.4(P-F).

S3. NMR spectra of some representative ME-FILs in CD_3OD

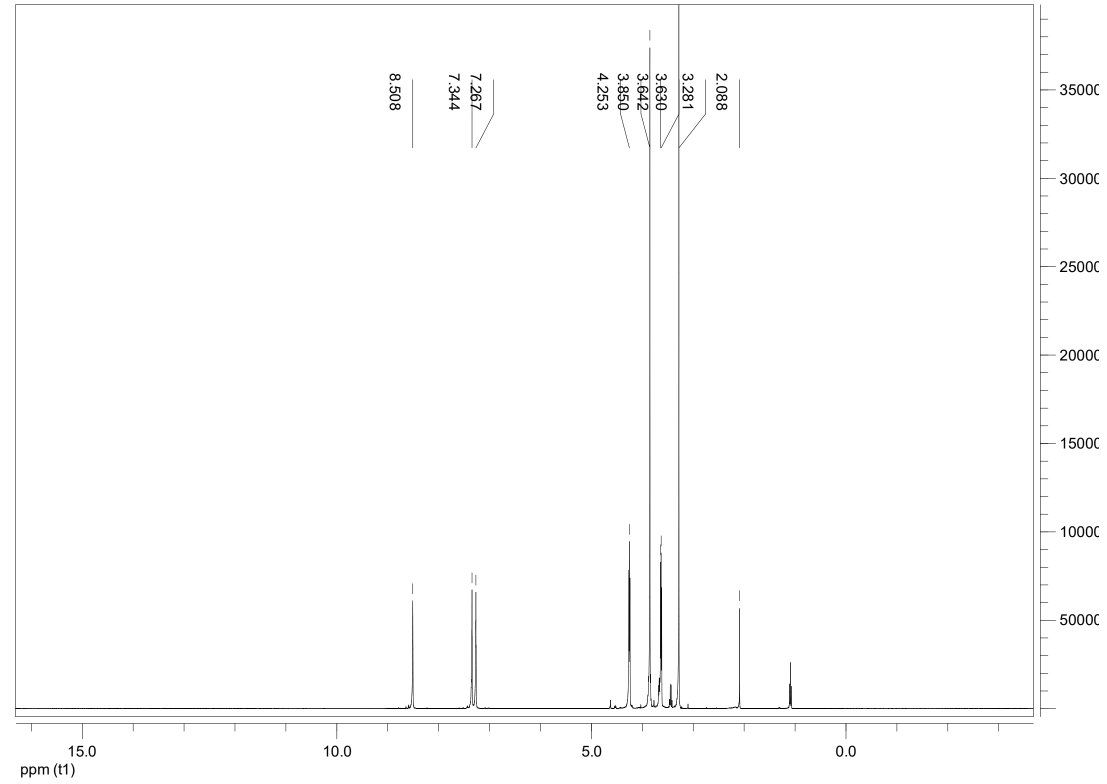

Figure *S1*. ^{1}H spectra of ME-FIL**1.**

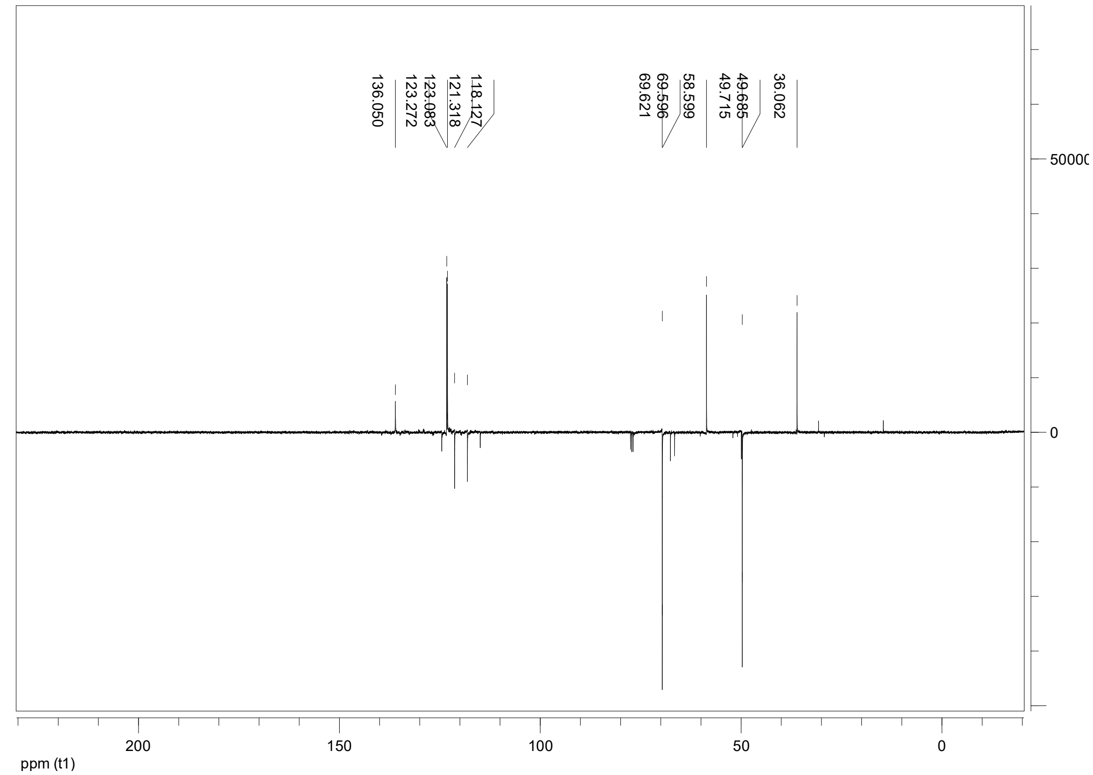

Figure *S2*. ^{13}C spectra of ME-FIL**1.**

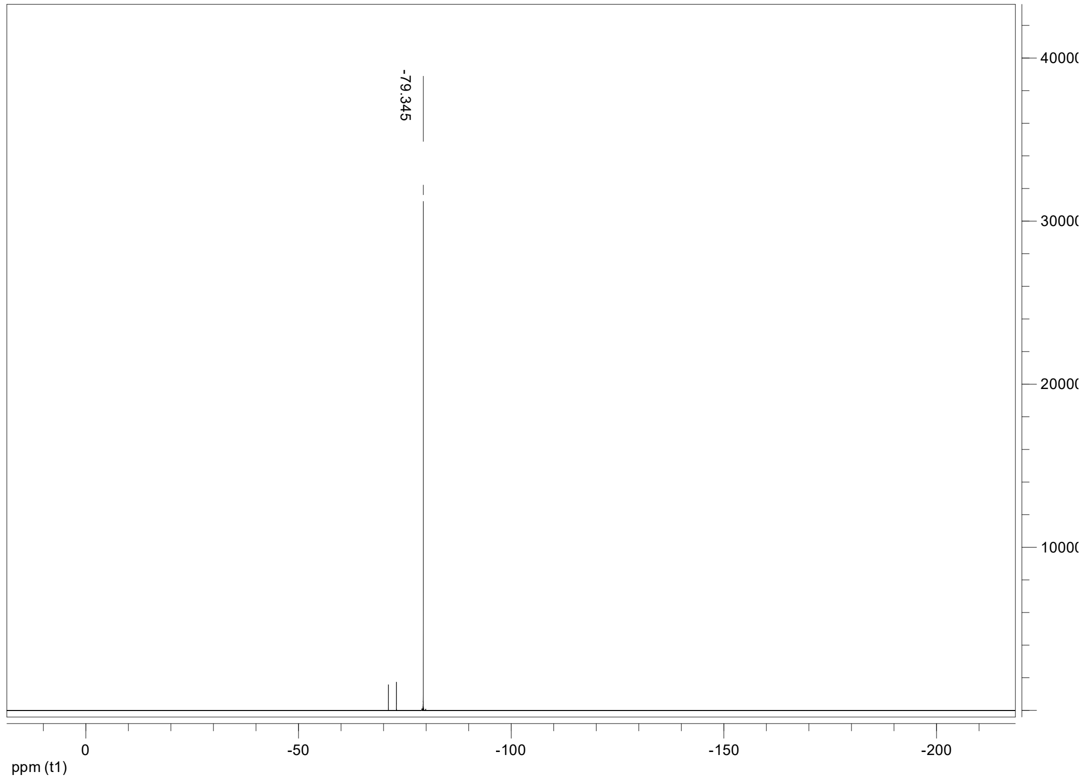

Figure *S3*. ^{19}F spectra of ME-FIL**1**.

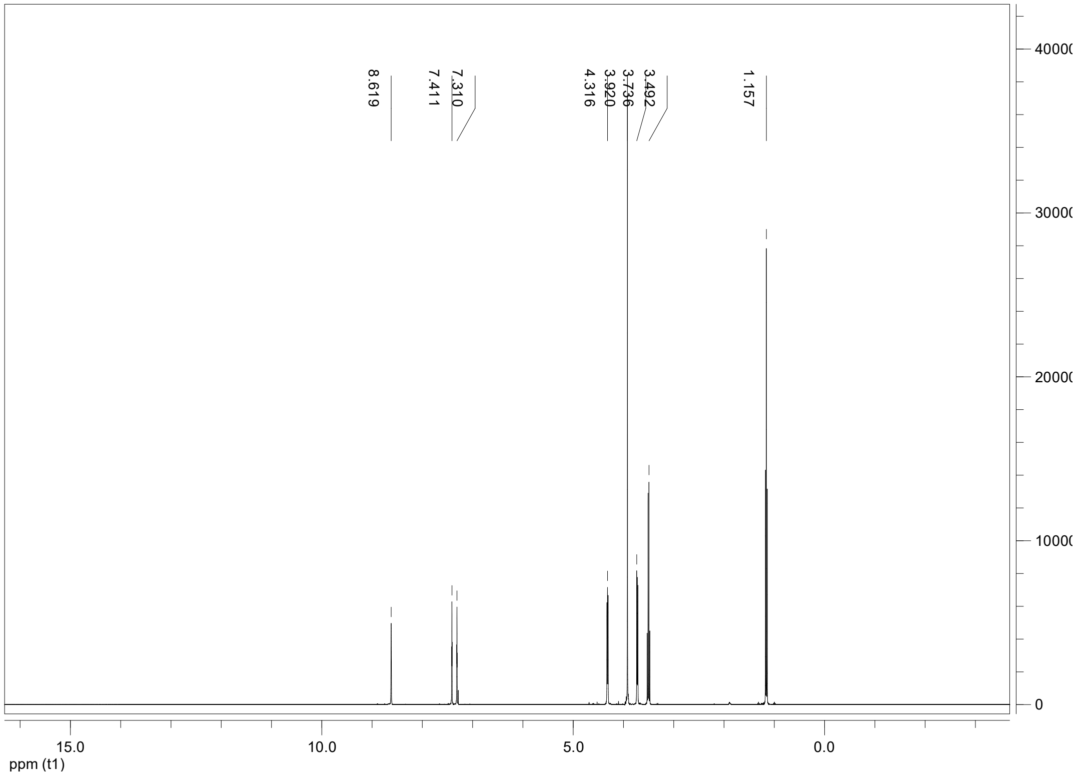

Figure *S4*. ^{1}H spectra of ME-FIL**2.**

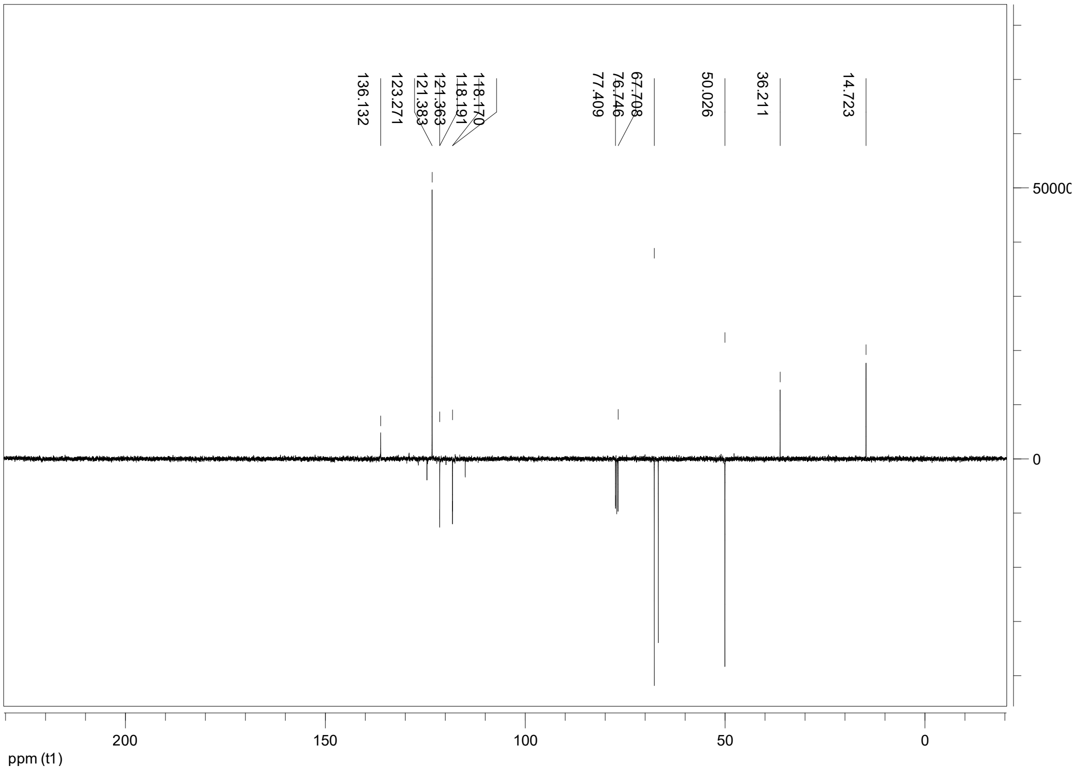

Figure *S5*. ^{13}C spectra of ME-FIL**2.**

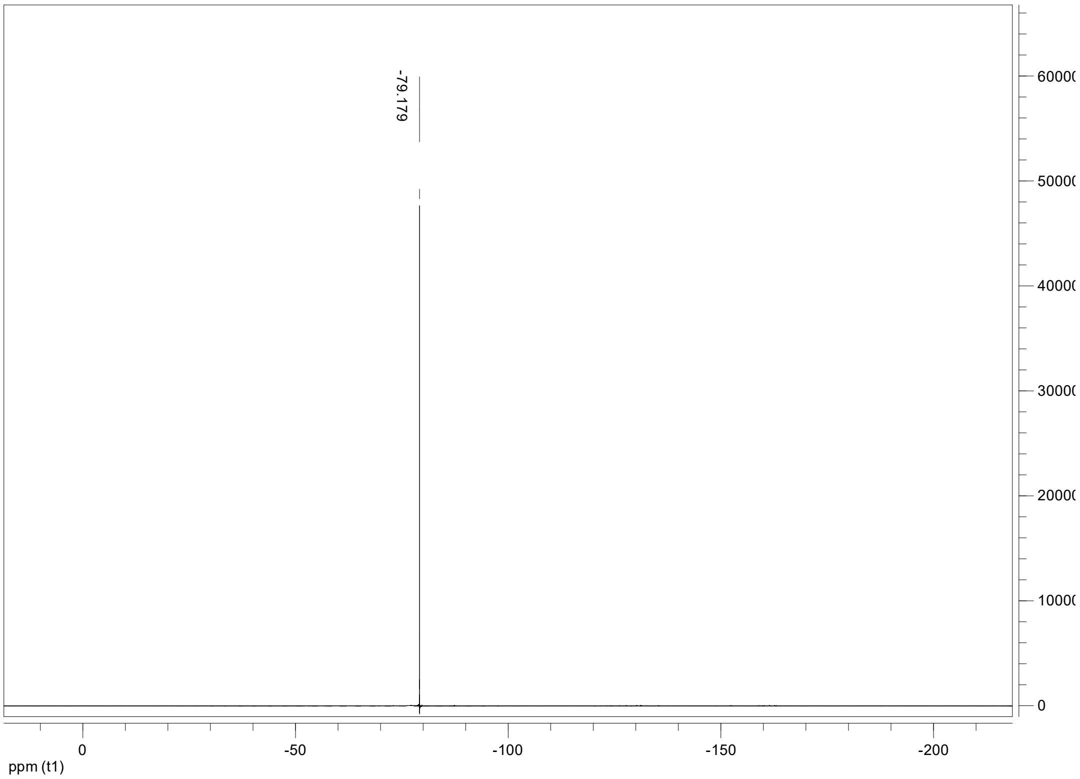

Figure *S6*. ^{19}F spectra of ME-FIL**2**.

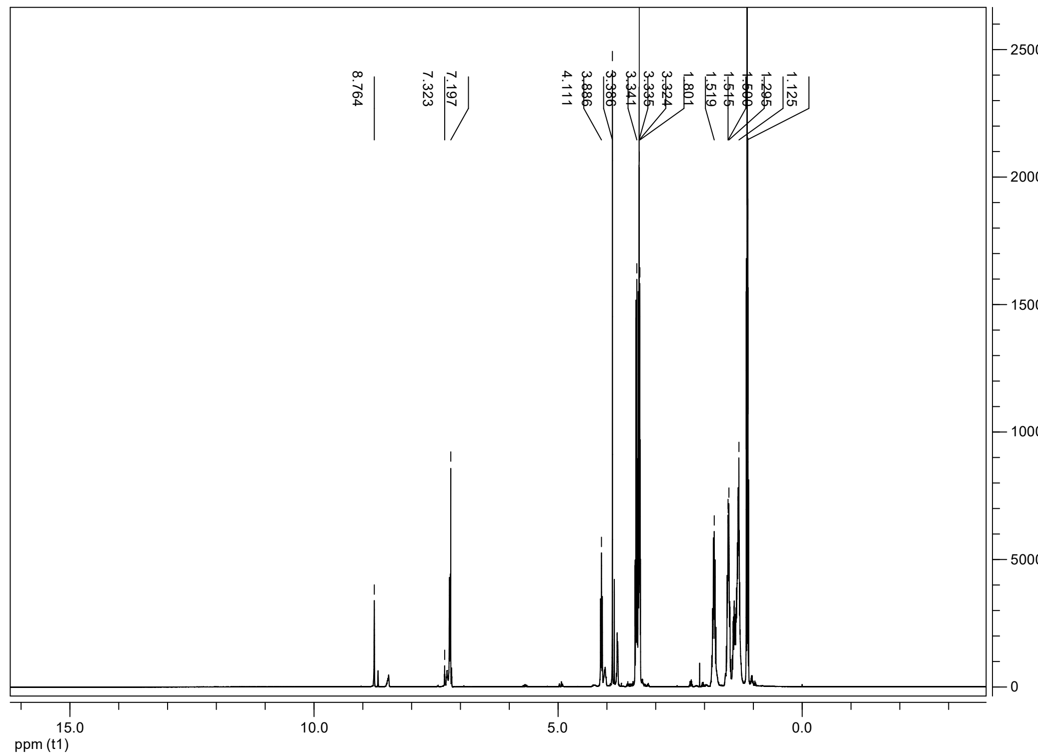

Figure *S7*. ^{1}H spectra of ME-FIL**3**.

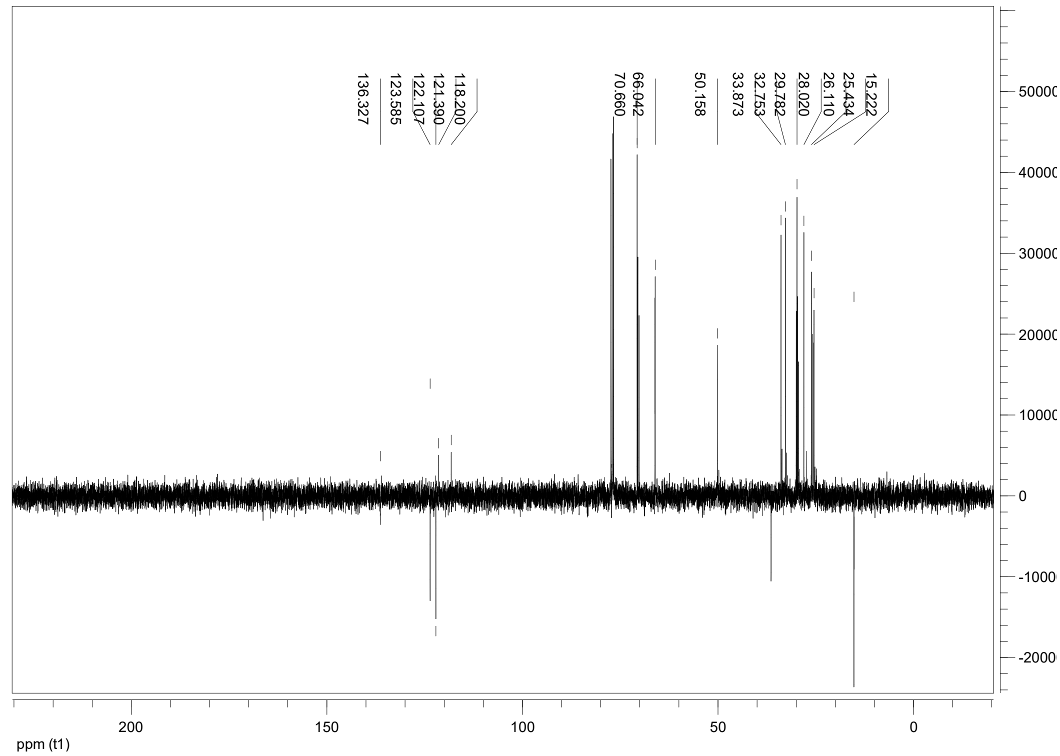

Figure *S8*. ^{13}C spectra of ME-FIL**3**.

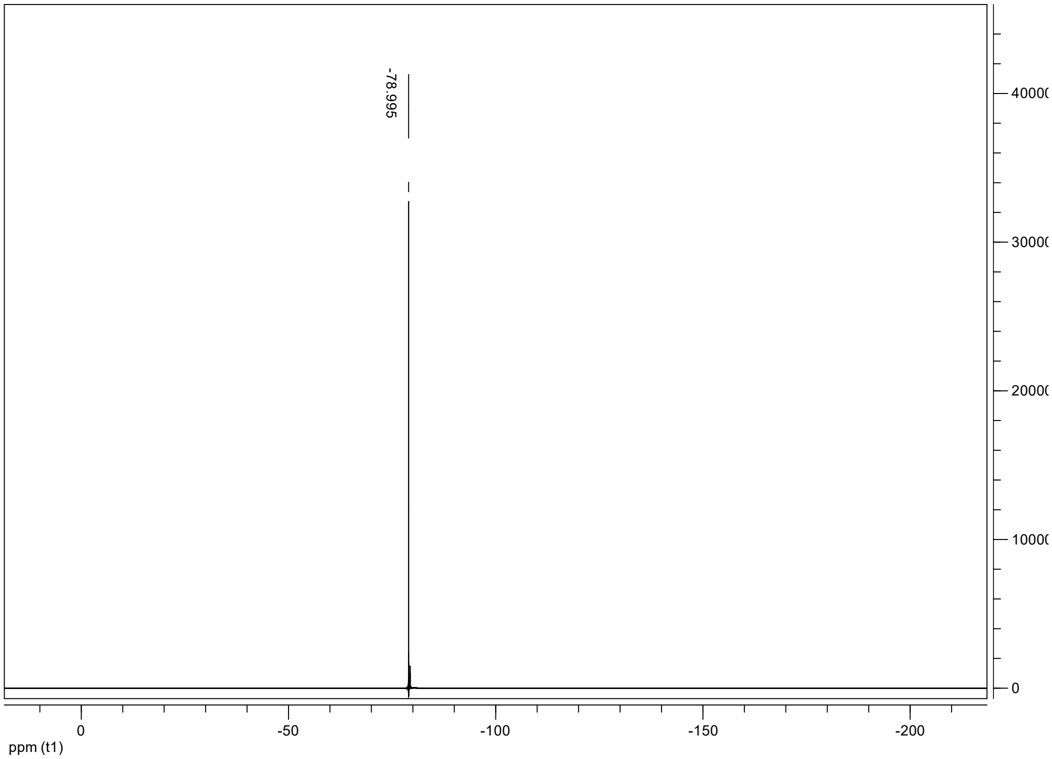

Figure *S9*. ^{19}F spectra of ME-FIL**3**.

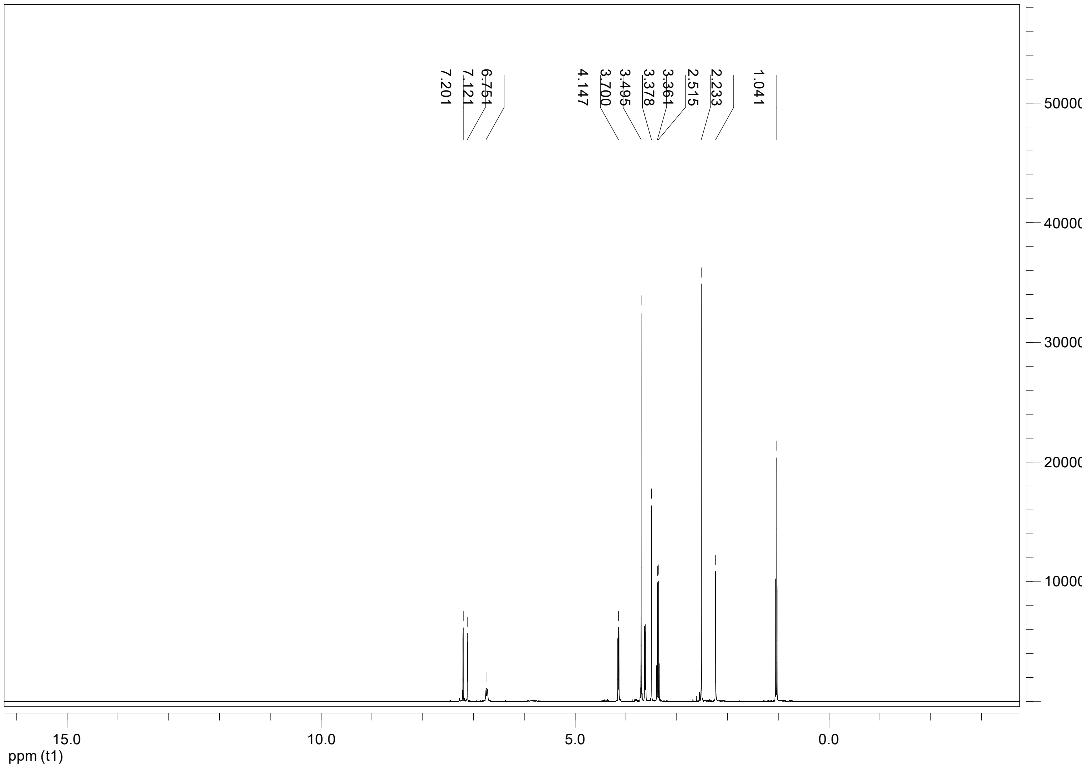

Figure *S10*. ^{1}H spectra of ME-FIL**4**.

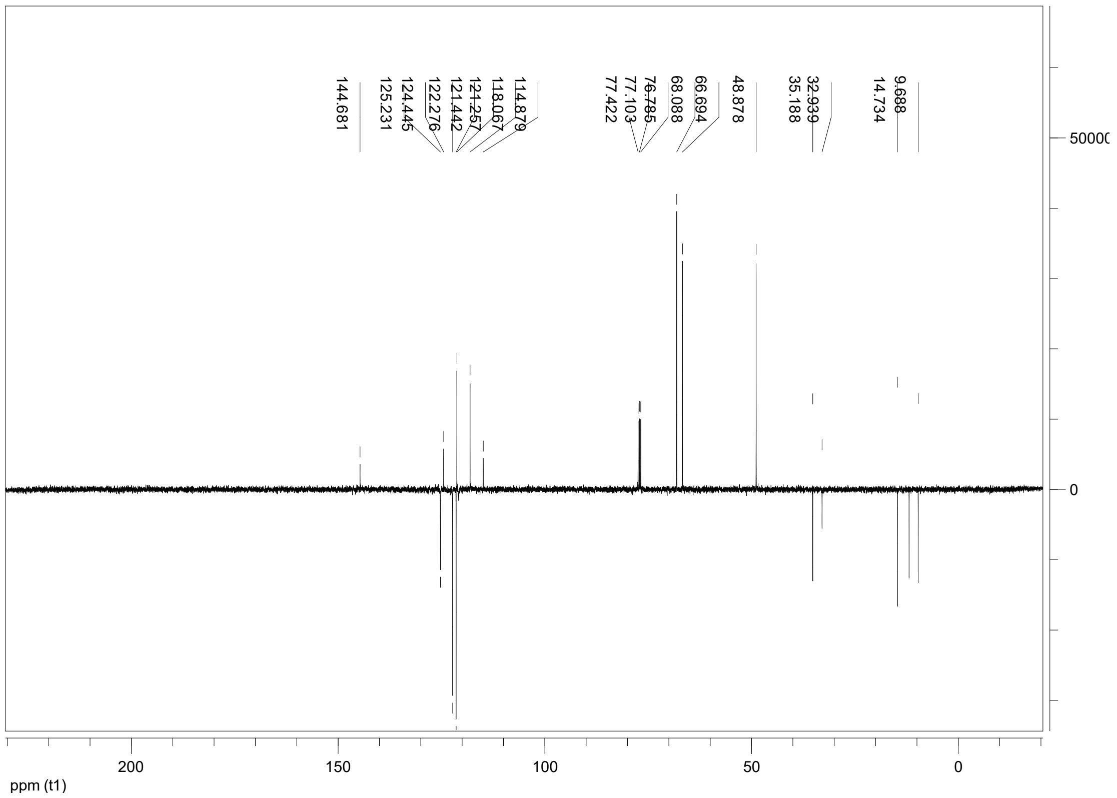

Figure *S11*. ^{13}C spectra of ME-FIL**4**.

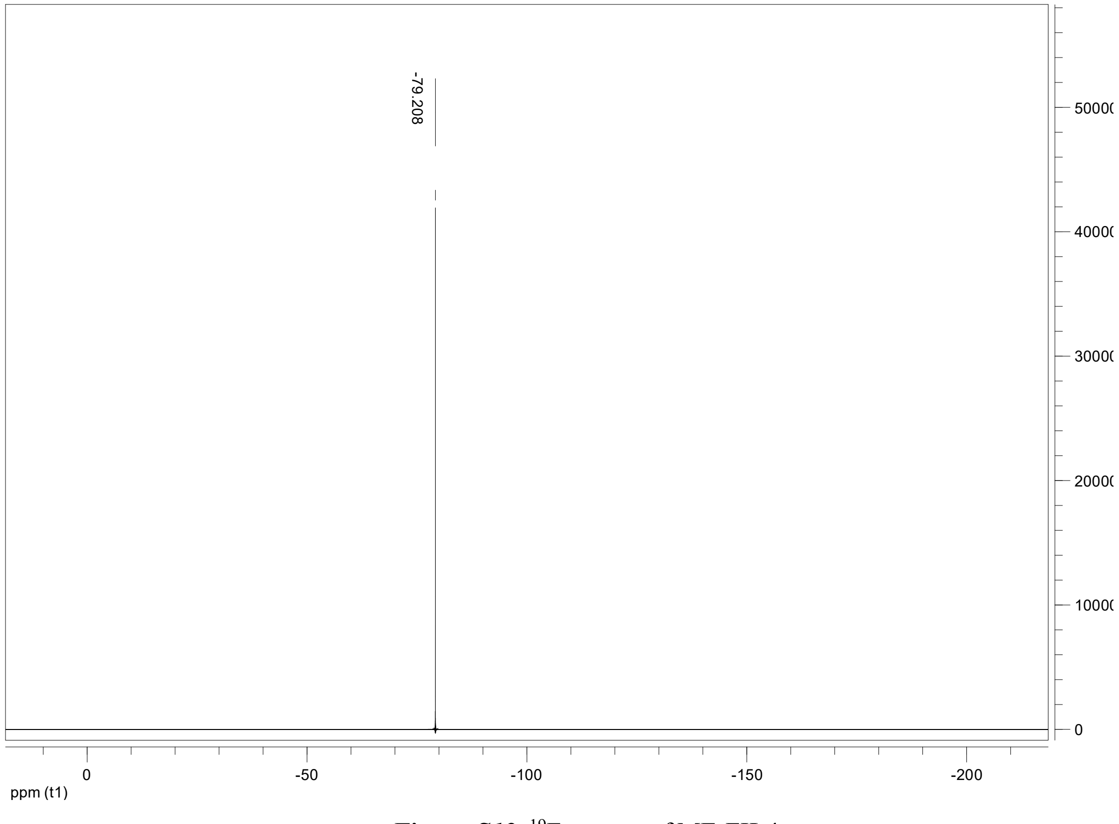

Figure *S12*. ^{19}F spectra of ME-FIL**4**.

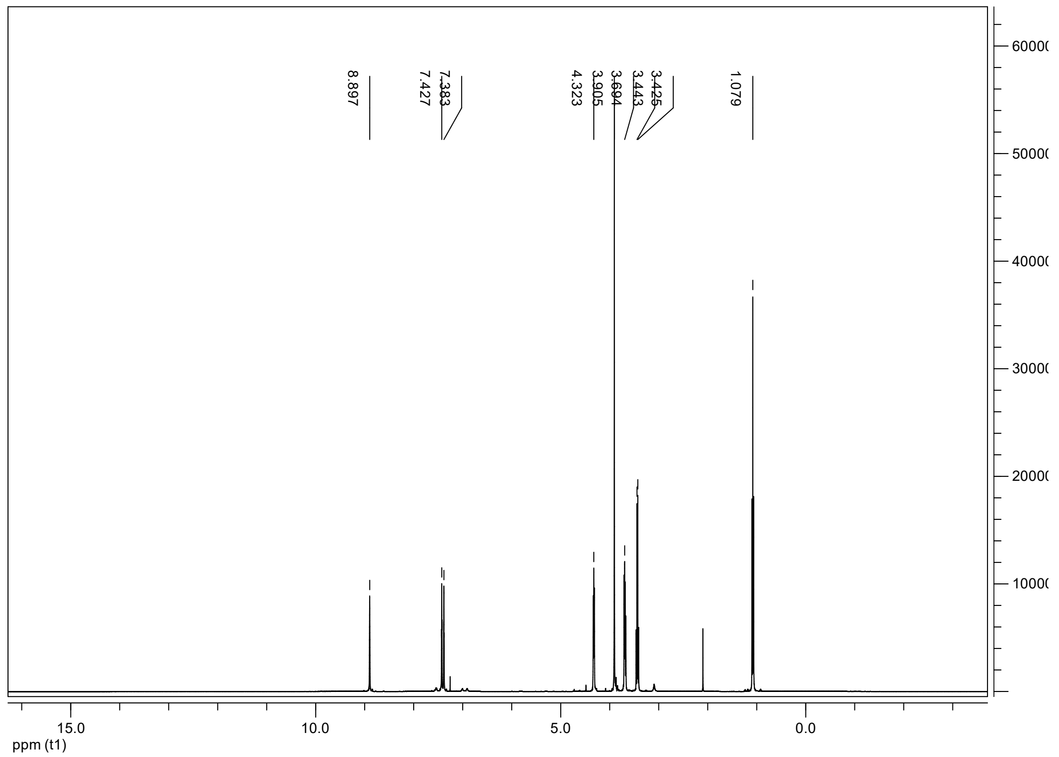

Figure *S13*. ^{1}H spectra of ME-FIL**6**.

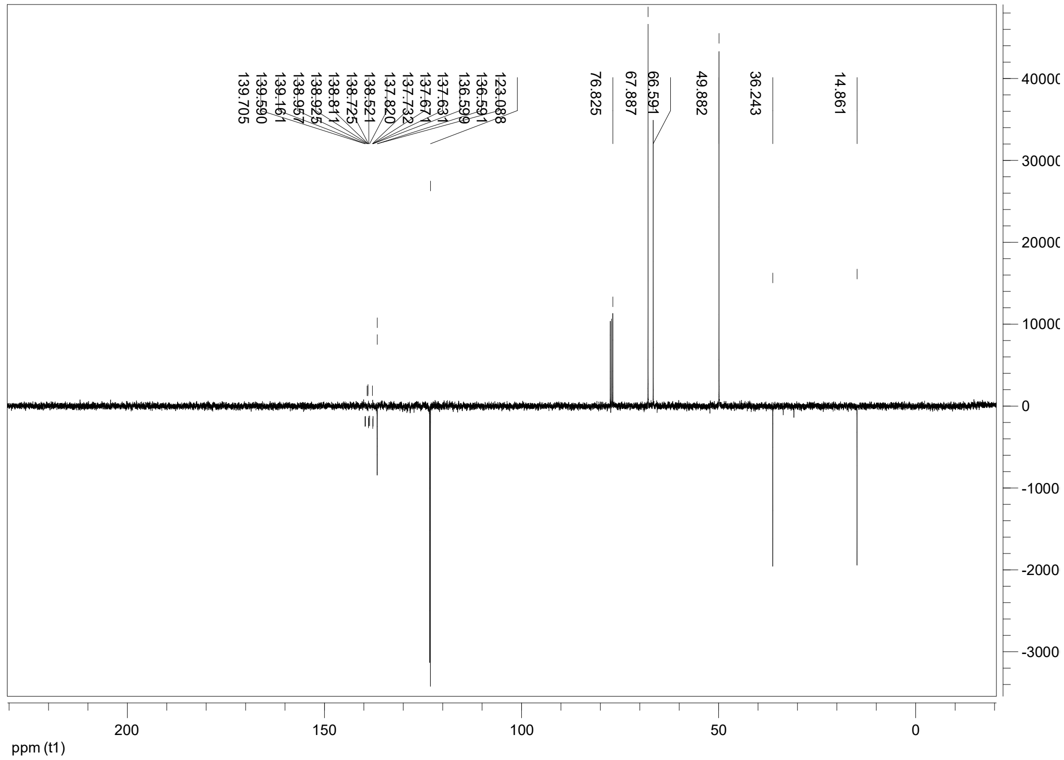

Figure *S14*. ^{13}C spectra of ME-FIL**6**.

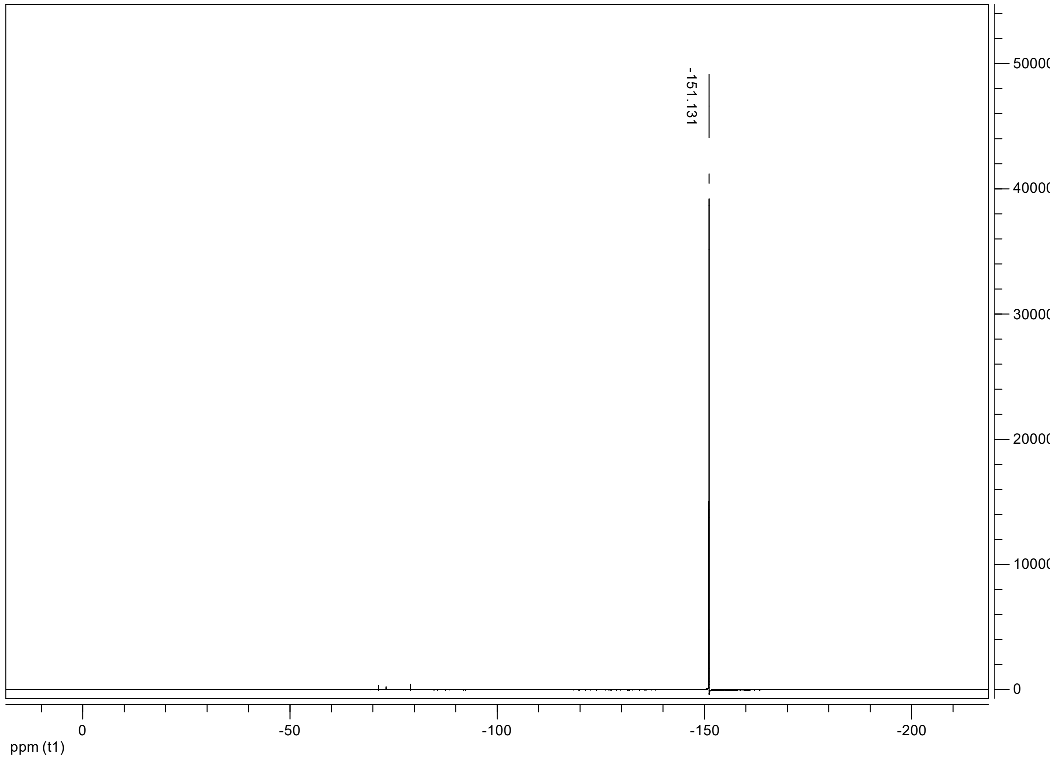

Figure *S15*. ^{19}F spectra of ME-FIL**6**.

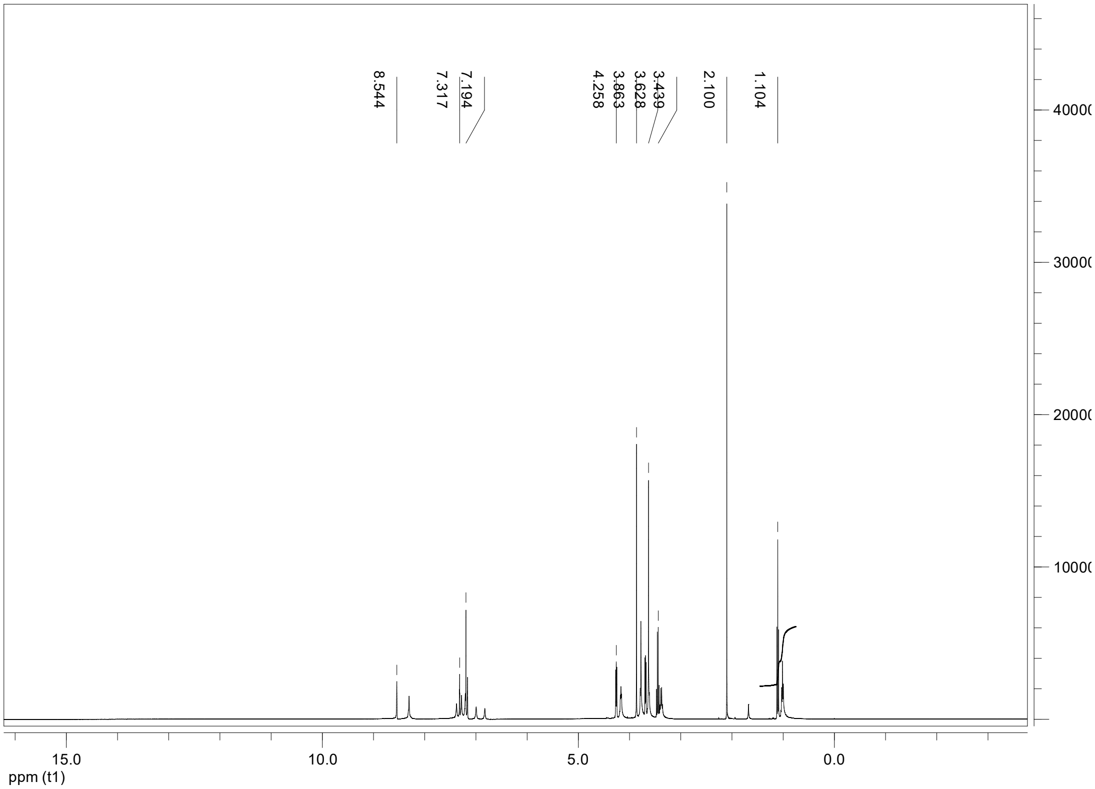

Figure *S16*. ^{1}H spectra of ME-FIL**7**.

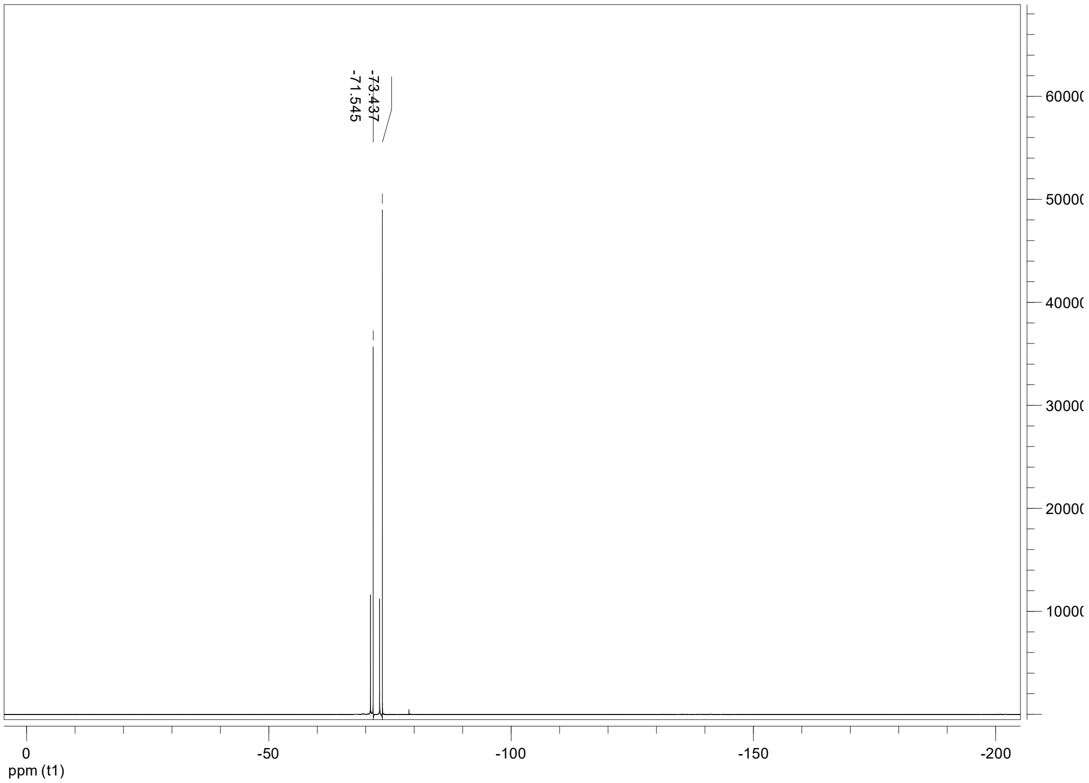

Figure *S17*. ^{19}F spectra of ME-FIL**7**.

S4. Mass Spectra of the typical ME-FILs

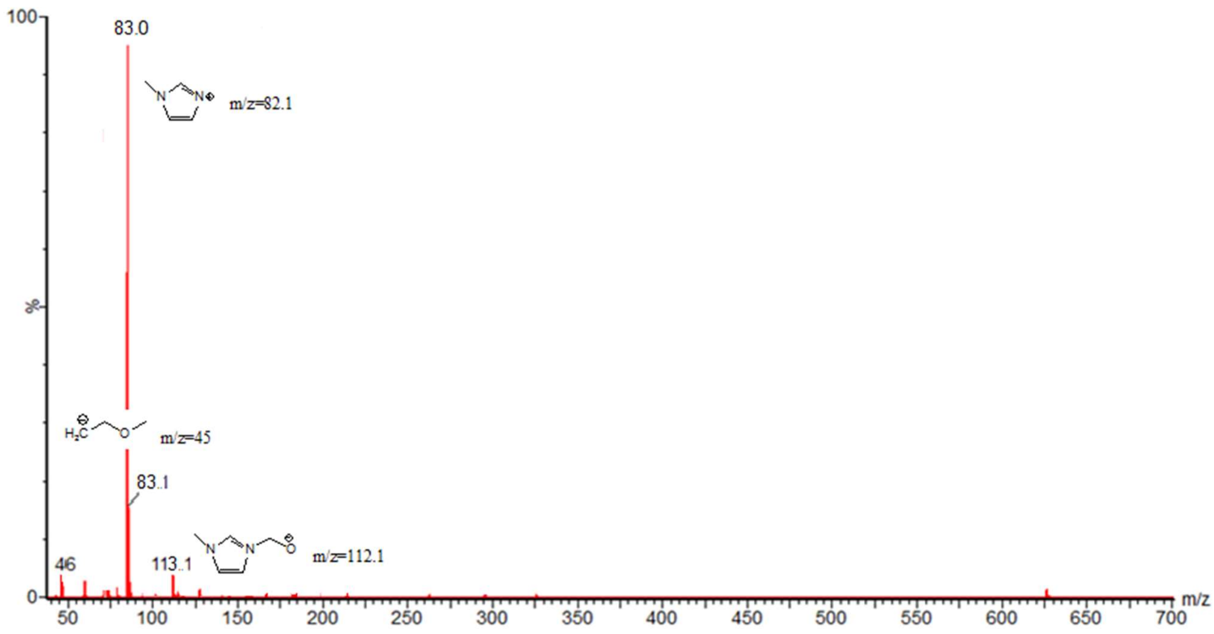

Figure *S18.* The cationic Mass spectrum of ME-FIL**1**.

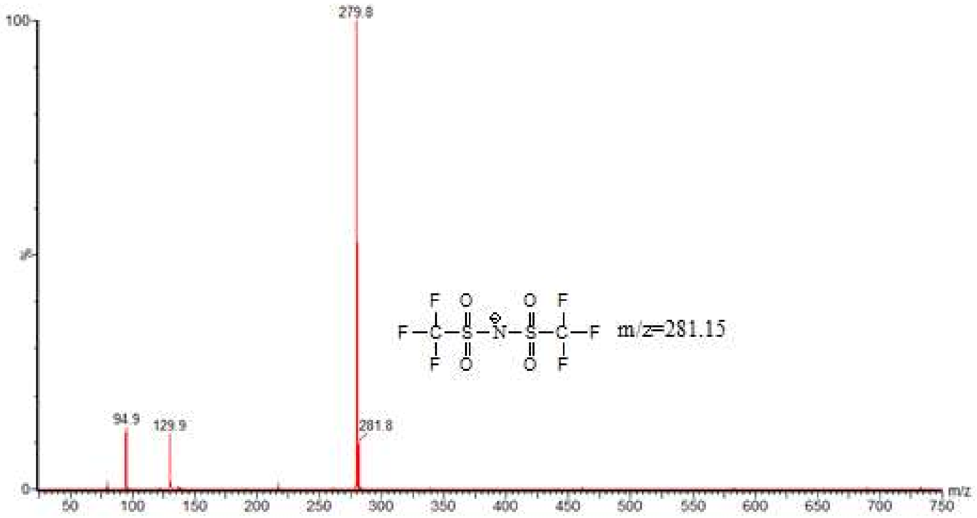

Figure *S19.* The anionic Mass spectrum of ME-FIL**1**.

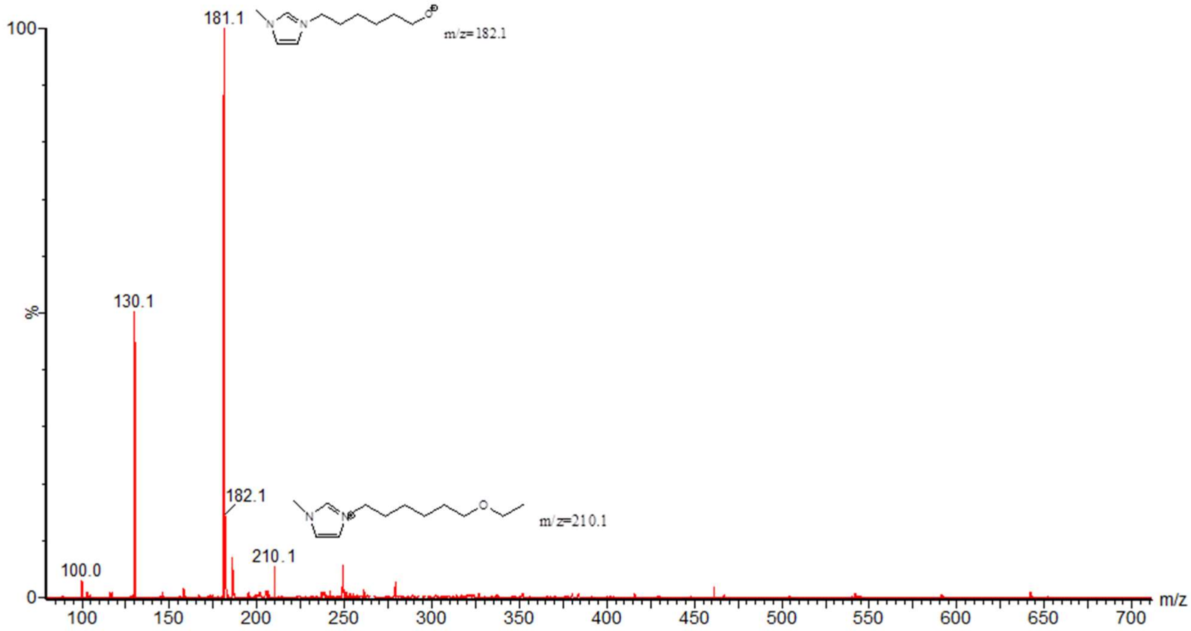

Figure S20. The cationic Mass spectrum of ME-FIL**3.**

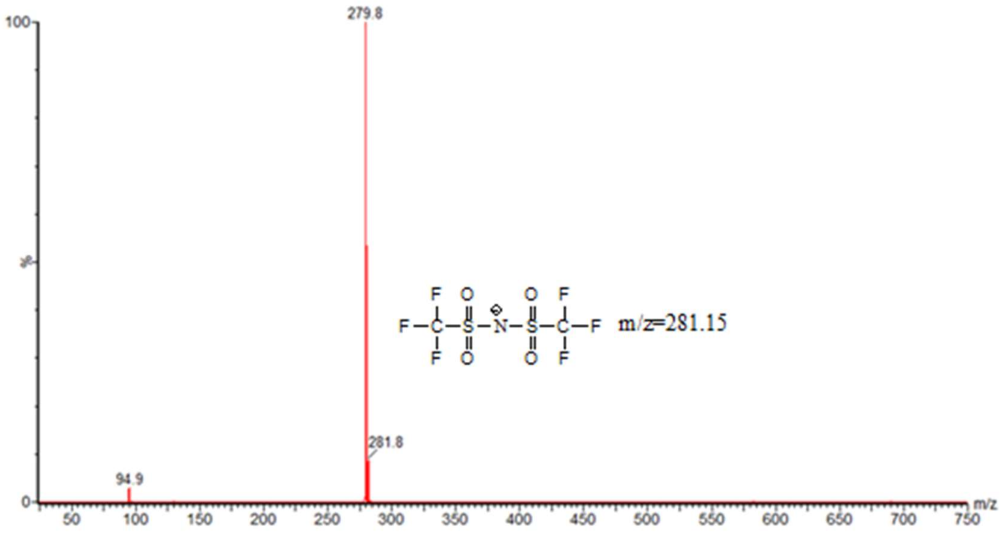

Figure ***S21*.** The anionic Mass spectrum of ME-FIL**3**.

S5. Water content of ME-FILs

The water content in all ME-FILs was quantified before each experiment using Karl-Fischer coulometric titration (C10SX from Mettler-Toledo). Before the titration, the ME-FILs was rotary evaporated in vacuum at 120 ºC for 24 h, and treated with anhydrous $CaCl_2$ pellets, then 3 g IL was chosen as titration samples (H_2O concentration detection limit = 4 ppm/0.3 mM).

**ME-FILs**	**1**	**2**	**3**	**4**	**5**	**6**	**7**
Water content	32 ppm	18 ppm	14 ppm	16 ppm	15 ppm	30×10^3 ppm	<100 ppm

***S6*. DSC plot of ME-FILs**

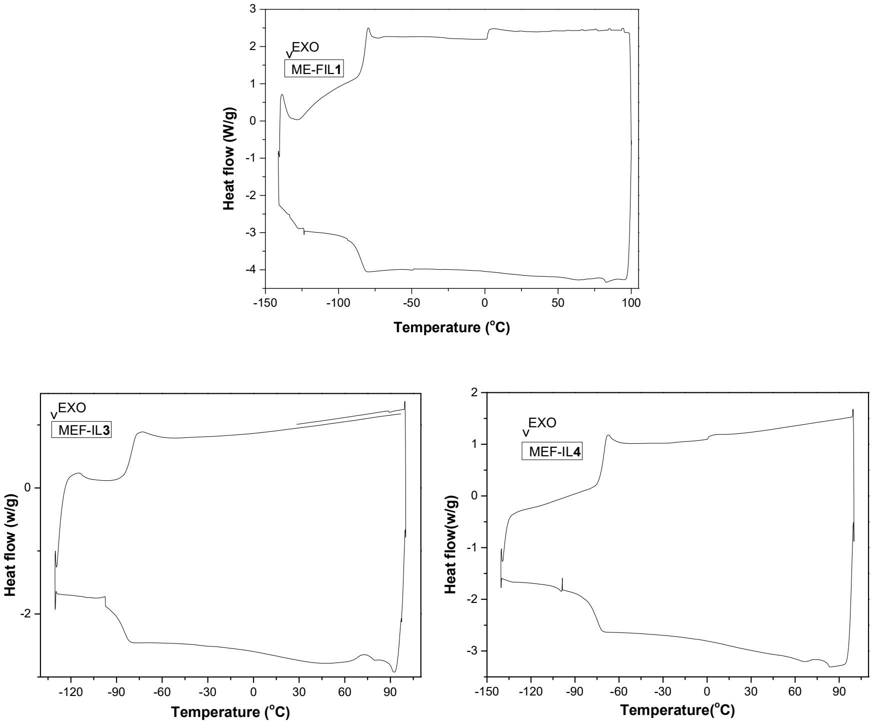

Figure *S22* DSC curves of MEF-IL**1**, **3**, and **4**.

S7. Cyclic voltammogram of ME-FILs

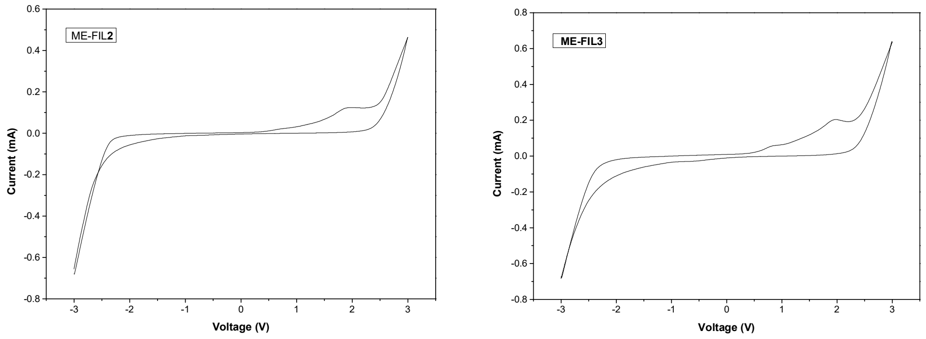

Figure *S23*. CV curves of MEF-IL**2** and **3**.

S8. Calculation of the heat capacity of ME-FILs

In a "three-step" method for the determination of the heat capacity of a material, the same temperature program must be applied to an empty sample pan, the sample, and the reference sample, both the sample and the reference sample were sealed in the alumium pans, respectively, as the empty pan, and heat capacities are calculated with the following equation[1,2].

$$C_{\mathrm{p,sample}} = \frac{\mathrm{Q_{sample}} - \mathrm{Q_{empty}}}{\mathrm{Q_{reference}} - \mathrm{Q_{empty}}} \frac{n_{\mathrm{reference}}}{n_{\mathrm{sample}}} C_{\mathrm{p,reference}}$$

where Q stands for the heat flow of the sample ($\mathrm{Q_{sample}}$), the reference sample ($\mathrm{Q_{reference}}$) or the empty pan ($\mathrm{Q_{empty}}$), while *n* describes the number of moles of sample (n_{sample}) or of the reference sample ($n_{\mathrm{reference}}$). This method uncertainty is about 13% [3].

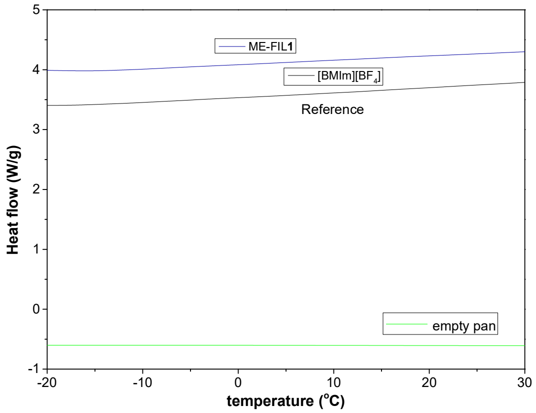

Figure *S24*. "Three-step" method for the determination of heat capacities.

In this work, the temperature program contains an isothermal phase of 15 min at starting temperature (-40 ºC) before the temperature is increased with a heating rate of 20 $\mathrm{Kmin^{-1}}$. Afterwards, the final temperature (40 ºC) is kept constant for 15 min. This procedure is illustrated in Figure *S20* for ME-FIL**1**. As showed in Figure *S20*, ME-FIL**1** is the tested sample, [BMIm][BF_4] IL is the reference, where $\mathrm{Q_{sample}}$=4.24 J/g, $\mathrm{Q_{reference}}$=3.69 J/g, $\mathrm{Q_{empty}}$=0.61J/g at 20 ºC, $\mathrm{Mr_{(ME\text{-}FIL1)}}$=407 g/mol, $\mathrm{Mr_{([BMIm][BF4])}}$=226.2 g/mol, $\mathrm{m_{sample}}$=15.019 mg, $\mathrm{m_{reference}}$=8.499 mg, $C_{\mathrm{p,reference}}$=1.6 J $\mathrm{K^{-1}g^{-1}}$ (Reference 4).

$$C_{\mathrm{p,sample}} = \frac{\mathrm{Q_{sample}}-\mathrm{Q_{empty}}}{\mathrm{Q_{reference}}-\mathrm{Q_{empty}}} \times \frac{\mathrm{n_{reference}}}{\mathrm{n_{sample}}} \times C_{\mathrm{p,reference}} = \frac{4.24- \ .61}{3.69- \ .61} \times \frac{8.499/226.2}{15.019/407} \times 1.6 = 1.2\ \mathrm{J\ K^{-1}g^{-1}}$$

Thus, $C_{\mathrm{p,m}}$=1.2×Mr=1.2×407=488.4 $\mathrm{JK^{-1}mol^{-1}}$.

References

1. Höhne, G.; Hemminger, W.; Flammersheim, H.-J. Differential Scanning Calorimetry, Springer-Verlag, Berlin, Heidelberg, New York, 1996.
2. Diedrichs, A.; and Gmehling, J. *Fluid Phase Equilibria.* 2006, **224**, 68.
3. Paulechka, Y. U. *J. Phys. Chem. Ref. Data.* 2010,39, 03310801.
4. Frez, C.; Diebold, G. J. *J. Chem. Eng. Data.* 2006, **51**, 1250.
